# The Impact of DRG Payments on Nutritional Therapy Costs for Gastric Cancer Surgery Patients

**DOI:** 10.3390/healthcare14101276

**Published:** 2026-05-08

**Authors:** Yuhan Wu, Hua Zhang, Yao Tang, Bo Xie, Xiao Zhang, Mo Liu, Qian Cao

**Affiliations:** 1 Department of Health Insurance, School of Public Health, Southeast University, Nanjing 210009, China; wuyuhan0120@163.com (Y.W.);; 2 Department of Psychology, Dornsife College of Letters, Arts and Science, University of Southern California, Los Angeles, CA 90089, USA; 3 Department of General Medicine, School of Medicine, Southeast University, Nanjing 210009, China; 4 Miller School of Albemarle, Charlottesville, VA 22903, USA

**Keywords:** DRG, gastric cancer, nutritional therapy, medical costs, interrupted time series analysis

## Abstract

**Objective:** This study examines the effect of DRG payment reform on nutritional therapy costs, hospitalization expenditures, and resource utilization among gastric cancer surgery patients using a quasi-experimental design. **Methods:** We conducted an interrupted time series analysis using inpatient data from a tertiary hospital in a major Chinese city between January 2018 and December 2024, including 761 gastric cancer surgery patients. Segmented regression models estimated changes in baseline trend, immediate level, and post-intervention slope for nutritional therapy expenditures, total hospitalization costs, surgical fees, and length of stay. Subgroup analyses were performed by type of medical insurance, and robustness checks were conducted. **Results:** Following DRG implementation, enteral nutrition costs showed a significant immediate increase (β_2_ = 395.703, *p* = 0.032) followed by a significant downward slope change (β_3_ = −7.778, *p* = 0.032). Total hospitalization costs demonstrated a significant immediate rise (β_2_ = 15,959.403, *p* = 0.019) and subsequent decline (β_3_ = −632.069, *p* < 0.001). Parenteral nutrition costs exhibited a significant immediate reduction (β_2_ = −2917.276, *p* = 0.001) without sustained trend changes. Surgical fees showed a significant downward baseline trend (β_1_ = −39.951, *p* < 0.001) and a significant upward slope change (β_3_ = 68.107, *p* < 0.001). Subgroup analyses revealed that policy effects were concentrated among patients with Urban Employee Basic Medical Insurance, with no significant effects observed for those with Urban and Rural Resident Basic Medical Insurance. Robustness checks confirmed the main findings. **Conclusions:** DRG payment reform significantly reshaped nutritional therapy and hospitalization expenditure patterns among gastric cancer surgery patients, with effects concentrated in the employee insurance population. The observed temporal pattern, characterized by an initial change followed by a gradual trend shift, suggests the need for continuous policy monitoring, insurance-tailored strategies, and refinement of nutritional support practices within DRG frameworks.

## 1. Introduction

To address rising healthcare costs, countries worldwide are actively advancing reforms in health insurance payment systems to enhance cost control and optimize the allocation of healthcare resources [1]. Among these reforms, the diagnosis-related group (DRG) payment model has emerged as a key mechanism. By grouping inpatient cases with similar clinical characteristics and expected resource use, DRGs replace retrospective fee-for-service reimbursement with prospectively determined bundled payments [2]. Under this model, hospitals retain savings when actual costs are below the fixed payment but bear financial losses when costs exceed the reimbursement rate. This creates incentives to shorten length of stay, reduce unnecessary services, standardize care pathways, and improve internal cost management, potentially encouraging a shift in provider behavior from volume expansion toward cost-efficient care delivery [3].

However, its implementation poses challenges in clinically complex cases that require multidisciplinary management and individualized treatment. On the one hand, the potential for inaccurate reimbursement in such cases may lead to insufficient economic compensation [4]. On the other hand, the strong cost-containment incentives inherent in DRGs may unintentionally reduce the provision of supportive therapies, such as nutritional support, which may have implications for patient outcomes [5]. Nutritional therapy costs therefore merit separate examination within DRG systems because they represent a clinically important but potentially vulnerable component of inpatient care [2]. Unlike major surgical procedures or high-cost drugs, nutritional support may be less visible in DRG grouping and payment weights, yet its underuse may be associated with poorer postoperative recovery [6].

This issue is particularly relevant in China, where DRG payment reform has been promoted as a major component of national medical insurance payment reform. Following the national CHS-DRG pilot initiated by the National Healthcare Security Administration, local governments gradually introduced DRG-based payment for inpatient care [7]. Nanjing, the setting of this study, officially implemented DRG payment reform on 1 January 2022 [8]. This local implementation occurred during China’s broader transition from itemized fee-for-service reimbursement toward prospective case-based payment, providing an institutional context for examining how DRG incentives may affect hospital cost structures and specific components of care.

Gastric cancer remains one of the most prevalent malignant tumors worldwide. According to the 2022 Global Cancer Statistics issued by the International Agency for Research on Cancer, gastric cancer ranked fifth in global cancer incidence, accounting for approximately 4.9% of all new cases [9]. In China, both incidence and mortality rates remain high; national statistics indicate that gastric cancer was the fifth most frequently diagnosed malignancy in 2022, representing 7.4% of new cancer cases [10]. Beyond its epidemiological burden, gastric cancer surgery is a typical high-cost inpatient service with relatively standardized surgical pathways but substantial heterogeneity in tumor stage, resection extent, perioperative complications, neoadjuvant or adjuvant treatment, and nutritional status [11,12]. These features make gastric cancer an appropriate condition for examining whether DRG-based cost control affects the allocation of resources for essential supportive therapies.

Nutritional therapy is particularly important in the clinical and economic management of gastric cancer patients. Malnutrition is one of the most critical factors affecting prognosis in this population, as it increases the risk of severe complications, reduces survival, and prolongs hospitalization, thereby exacerbating both the clinical and financial burdens on patients [13,14,15]. To address this issue, standardized principles and approaches for nutritional therapy were proposed as early as 2013 [16]. The 2025 ESPEN guideline on clinical nutrition in surgery further states that nutritional therapy is mandatory for surgical patients at nutritional risk, particularly those undergoing upper gastrointestinal surgery [9]. Recent evidence from randomized trials and meta-analyses also suggests that nutritional interventions may improve nutritional and immune indicators, reduce infectious complications, shorten hospitalization, and lower medical costs in selected gastric cancer patients [4,17,18]. Nevertheless, the implementation of nutritional support in clinical practice remains suboptimal. Under growing pressure to control healthcare expenditures, hospitals often prioritize direct treatment spending, frequently at the expense of supportive care such as nutritional therapy. Moreover, malnourished patients tend to incur higher medical costs due to additional treatments and extended hospital stays [19]. While DRG-based payment reforms impose new pressures on nutritional management in gastric cancer, they may also be associated with more standardized and efficient care delivery. Thus, the net impact of DRG implementation on nutritional therapy remains uncertain.

Although DRG-based payment reform has been widely implemented to improve efficiency and control costs, existing evidence has primarily focused on overall hospitalization expenditures, length of stay, and major cost components such as drugs and consumables [20,21,22,23]. Recent studies have further shown that DRG reform can alter cost structures and reduce inpatient expenditures, particularly in surgical populations, but these analyses largely overlooked the role of nutritional therapy [24,25]. Even internationally, concerns regarding nutritional therapy reimbursement under DRG were raised as early as the initial phases of DRG adoption [26], but focused investigations have only recently emerged and remain insufficient [13,27]. This gap is especially relevant in the Chinese context: while Chinese scholars have conducted extensive research on the economic and clinical aspects of nutritional therapy [28,29,30,31,32], no study has evaluated nutritional support within the framework of DRG payment reform. To address these gaps, this study applies an interrupted time-series design to examine the dynamic impact of DRG reform on nutritional therapy costs among gastric cancer surgery patients.

Given these gaps, this study aims to examine how the DRG-based payment model affects nutritional therapy expenditures among gastric cancer surgery patients. By analyzing real-world expenditure patterns before and after DRG implementation, we seek to clarify how supportive therapies are utilized within the broader framework of health insurance payment reform. This inquiry is crucial for guiding cancer care policy. This study contributes policy-relevant evidence in three ways: first, it helps assess whether current DRG grouping and payment weights adequately reflect the resource needs of nutritionally vulnerable cancer surgery patients; second, it informs the design of monitoring indicators related to nutritional care, such as nutritional screening and appropriate nutritional support, under case-based payment; and third, it provides evidence for hospitals and payers seeking to improve cost efficiency while ensuring adequate provision of supportive care, as it relates to balancing healthcare system sustainability and patient-level considerations.

## 2. Methodology

### 2.1. Data Source

Nanjing City officially implemented its DRG payment reform on 1 January 2022. Taking advantage of this policy shift as a natural experiment, we identified all gastric cancer surgery patients who received nutritional therapy at the study hospital between 1 January 2018 and 31 December 2024.

#### 2.1.1. Inclusion Criteria

This study adopted the following inclusion criteria, developed in accordance with the “2022 Nanjing Interim Measures for DRG Point-Based Payment in Basic Medical Insurance.” Eligible patients were required to have a primary diagnosis of gastric cancer (ICD-10 code C16) based on the CHS-DRG grouping scheme (Version 1.1). In addition, patients had to (1) undergo a surgical procedure, (2) be 18 years of age or older, (3) have their medical expenses settled between 1 January 2018, and 31 December 2024, and (4) have received nutritional therapy, including enteral or parenteral nutritional preparations.

#### 2.1.2. Exclusion Criteria

Patients were excluded if their medical records met any of the following conditions: (1) missing data for key analytical variables; (2) the presence of extreme statistical outliers that could not be corrected through validation; or (3) clinically implausible or logically inconsistent information, such as documented surgical procedures without corresponding treatment costs or a recorded length of stay of less than one day.

After applying the inclusion and exclusion criteria, a total of 761 cases were retained for analysis, including 261 patients treated before DRG implementation and 500 patients treated in the post-reform period. With a total of 84 time points, on average each time point included approximately 9–10 cases, likely affording sufficient data density for time series estimation.

### 2.2. Variable Selection and Processing

This study selected enteral nutrition costs, parenteral nutrition costs, duration of nutritional therapy, surgical fees, total hospitalization costs, and length of stay as outcome variables to comprehensively assess both the economic burden of nutritional support and the overall healthcare resource utilization among gastric cancer surgery patients.

Enteral nutrition cost was defined as the total expenditure on all enteral nutritional formulas administered during hospitalization. Parenteral nutrition cost represented the total spending on intravenous nutritional solutions and related medications.

The duration of nutritional therapy was measured as the number of consecutive days between the first and last administration of any nutritional supplement, serving as an indicator of the intensity and continuity of nutritional support.

To examine the broader influence of the DRG payment reform, we also analyzed surgical fees, total hospitalization costs, and length of stay, allowing for an evaluation of its impact on overall healthcare resource consumption.

To account for inflation, all nominal costs, including enteral nutrition, parenteral nutrition, surgical fees, and total hospitalization, were converted into constant 2022 values using the consumer price index (CPI). The specific adjustment formula is as follows:

(1) $$Adjusted\;Cost\;Indicator\;=\;\frac{Original\;Cost\;Indicator}{{CPI}_{Base\;Year}}\times {CPI}_{2022}$$

The CPI data used for cost adjustment were sourced from the China Statistical Yearbook 2024, published by the National Bureau of Statistics [27].

To minimize potential confounding effects, the analysis incorporated multiple control variables, including patient age, gender, type of medical insurance, preoperative prealbumin, type of surgery, and preoperative body mass index. These variables were selected to account for clinical and demographic differences that may influence both treatment decisions and healthcare expenditures. Specifically, preoperative prealbumin and body mass index were included as control variables because they serve as key indicators of patients’ preoperative nutritional status, which may influence both treatment decisions and subsequent healthcare expenditures. In addition, key variables required for the interrupted time series analysis were constructed, including a continuous time variable (t), a policy indicator (post), and an interaction term (post trend) to capture changes in trend following policy implementation. The definitions and measurements of the study variables are presented in Table 1.

### 2.3. Analysis Strategy

All statistical analyses were performed using Stata SE 16 (StataCorp LLC, College Station, TX, USA). Continuous variables were presented as median (interquartile range) and compared between groups using the Mann–Whitney U test. Categorical variables were summarized as frequencies and percentages, with comparisons made using the chi-square test.

To evaluate the effect of the DRG payment reform on nutritional therapy expenditures among gastric cancer surgery patients, this study employed an ITS design, a robust quasi-experimental method commonly used in health policy analysis. The model was specified as follows:

(2) $$Y_{t}=\beta_{0}+\beta_{1}T_{t}+\beta_{2}P_{t}+\beta_{3}(T_{t}\times P_{t})+\gamma X_{t}+\varepsilon_{t}$$

In this model, *Yₜ* represents the outcome variable measured at time point t. The variable *Tₜ* denotes time, recorded in monthly intervals. The parameter *β*_0_ reflects the baseline level of the outcome before the policy implementation, while *β*_1_ captures the pre-intervention trend. The coefficient *β*_2_ estimates the immediate change in the outcome level following the intervention, and *β*_3_ represents the change in the slope post-intervention, indicating the sustained effect of the policy over time. The dummy variable *Pₜ* differentiates the pre-intervention period (coded as 0) from the post-intervention period (coded as 1). *X_t_* denotes a vector of control variables, including age, gender, type of medical insurance, preoperative prealbumin, type of surgery, and preoperative body mass index. Finally, *εₜ* denotes the random error term.

The analysis utilized a segmented regression approach within the ITS framework. Statistical significance of the policy intervention was assessed by hypothesis testing of the regression coefficients, focusing on changes in trends of the outcome measures following the implementation of the DRG system. Heteroscedasticity-robust standard errors (Huber–White) were applied to all regression models to ensure valid inference. In addition, we formally tested for serial correlation in the residuals using the Durbin–Watson (DW) test and Breusch–Godfrey (BG) test. Where necessary, Newey–West standard errors were considered to ensure robustness of statistical inference.

To explore potential heterogeneity in policy effects across patient populations, we conducted subgroup analyses stratified by type of medical insurance. Patients were categorized into two groups: Urban Employee Basic Medical Insurance and Urban and Rural Resident Basic Medical Insurance. The interrupted time series model was re-estimated separately for each subgroup, maintaining the same specification as the primary analysis, including the same set of control variables (age, sex, preoperative prealbumin, body mass index, and type of surgery). The coefficients for baseline trend, level change, and slope change were reported for each subgroup.

To assess the robustness of our main findings, we performed two sensitivity analyses. First, we conducted a placebo test by shifting the policy implementation date to January 2023, a time point at which no actual policy change occurred. If the estimated effects under this false specification remained significant, it would suggest that the main findings may reflect secular trends rather than the true policy impact. Second, we excluded the three-month period immediately following policy implementation (January to March 2022) to examine whether the initial implementation phase—during which clinical practices may have been adjusting—disproportionately influenced the estimates. The ITS model was then re-estimated on the remaining observations.

### 2.4. Assumptions of the ITS Design

The validity of the interrupted time series design relies on several key identification assumptions. First, no other major policy interventions affecting nutritional therapy or hospital expenditures were implemented during the study period that could systematically coincide with the DRG reform. According to local policy records, no concurrent reforms specifically targeting nutritional therapy reimbursement or surgical care were introduced in the study hospital during 2018–2024.

**Figure 1:**
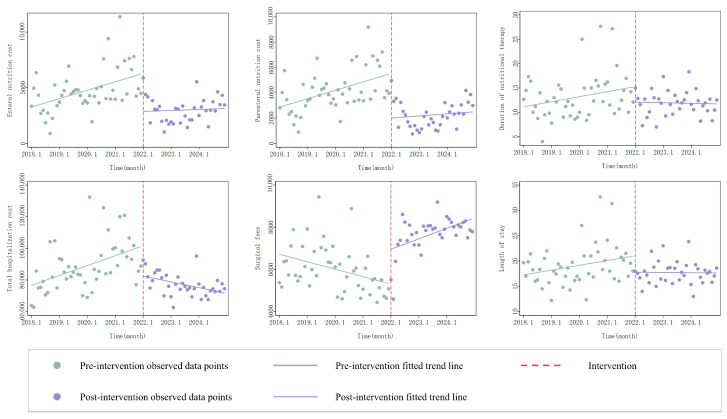
Interrupted time series analysis of outcome variables before and after DRG implementation.

Second, the pre-intervention trend is assumed to be stable and predictive of the counterfactual post-intervention trajectory in the absence of the reform. In this study, the pre-policy period spans 48 monthly observations, allowing for reliable estimation of baseline trends. Visual inspection of the time series plots (Figure 1) and statistically significant or stable β_1_ estimates support the assumption of a stable pre-intervention trend.

Third, the composition of the study population is assumed to remain relatively stable over time. To mitigate potential confounding, we adjusted for observable patient characteristics, including age, sex, insurance type, nutritional status, and surgery type.

## 3. Results

### 3.1. Basic Information of Included Patients

The final analytical sample included 761 patients, with 261 in the pre-intervention cohort and 500 in the post-intervention cohort. The majority of patients were male (549, 72.1%), and 558 patients (73.3%) were aged 60 years or above. Urban–Rural Resident Basic Medical Insurance was the predominant payment method, covering 674 cases (88.6%). A detailed breakdown of patient characteristics is presented in Table 2.

Baseline comparisons of continuous variables between the pre- and post-intervention groups are shown in Table 3. Age, body mass index, and preoperative prealbumin levels were comparable between the two groups, with all variables reported as median (interquartile range).

Table 4 summarizes the comparative analysis of outcome variables before and after DRG implementation using the Mann–Whitney U test. Following DRG implementation, enteral nutrition costs increased significantly from 403.10 (156.54, 708.97) to 610.49 (167.79, 951.91); Z = −3.785, *p* < 0.001. In contrast, parenteral nutrition costs decreased significantly from 3353.21 (2109.09, 5385.18) to 1827.31 (878.64, 3165.62); Z = 9.520, *p* < 0.001. The duration of nutritional therapy also showed a statistically significant reduction, decreasing from 11.0 days (8.0, 15.0) to 10.0 days (7.0, 14.0); Z = 2.053, *p* = 0.040. Total hospitalization costs decreased significantly from 81,987.67 (68,938.50, 96,668.36) to 74,014.10 (65,880.06, 83,041.13); Z = 5.333, *p* < 0.001. Surgical fees increased significantly from 5807.63 (4975.61, 6914.57) to 7798.62 (6874.84, 8323.69); Z = −12.625, *p* < 0.001. Length of stay decreased significantly from 17.0 (14.0, 21.0) to 16.0 (14.0, 20.0); Z = 2.182, *p* = 0.029.

### 3.2. Trends in Nutritional Therapy Costs for Gastric Cancer Surgery Patients Before and After DRG Payment Reform

The interrupted time series analysis results are summarized in Table 5 and illustrated in Figure 1.

Enteral nutrition costs exhibited a significant upward baseline trend before the policy intervention (β_1_ = 5.803, *p* = 0.027). Immediately following the introduction of the DRG payment system, there was a significant increase in the level of enteral nutrition expenditure (β_2_ = 395.703, *p* = 0.032), which was then followed by a significant downward slope change (β_3_ = −7.778, *p* = 0.032).

Parenteral nutrition costs showed no significant baseline trend (β_1_ = 22.984, *p* = 0.188), but a significant immediate level decrease was observed at policy implementation (β_2_ = −2917.276, *p* = 0.001), with no significant subsequent slope change (β_3_ = −0.131, *p* = 0.995).

For the duration of nutritional therapy, no significant baseline trend (β_1_ = 0.055, *p* = 0.208), level change (β_2_ = 0.216, *p* = 0.925), or slope change (β_3_ = −0.053, *p* = 0.322) was detected.

Total hospitalization costs exhibited a distinct pattern: a significant upward baseline trend (β_1_ = 431.584, *p* = 0.003), a significant immediate level increase at policy implementation (β_2_ = 15,959.403, *p* = 0.019), followed by a significant downward slope change (β_3_ = −632.069, *p* < 0.001).

Surgical fees showed a significant downward baseline trend (β_1_ = −39.951, *p* < 0.001), a marginally significant level decrease (β_2_ = −1442.363, *p* = 0.085), and a significant upward slope change (β_3_ = 68.107, *p* < 0.001).

Length of stay did not exhibit any statistically significant baseline trend (β_1_ = 0.060, *p* = 0.147), level change (β_2_ = −0.632, *p* = 0.762), or slope change (β_3_ = −0.043, *p* = 0.386).

### 3.3. Subgroup Analysis by Type of Medical Insurance

To examine whether the impact of the DRG payment reform varied across insurance schemes, we conducted stratified interrupted time series analyses for patients covered by Urban and Rural Resident Basic Medical Insurance (Table 6) and Urban Employee Basic Medical Insurance (Table 7).

Among patients with resident insurance, the DRG policy exhibited limited effects across most outcome measures. Total hospitalization costs showed a significant upward baseline trend (β_1_ = 872.634, *p* = 0.010), but no significant immediate level change (β_2_ = −101,551.449, *p* = 0.421) or subsequent slope change (β_3_ = 1228.840, *p* = 0.610) was detected. Similarly, surgical fees displayed a significant downward pre-policy trend (β_1_ = −57.025, *p* = 0.010), with no significant changes at or after implementation. For enteral nutrition costs, parenteral nutrition costs, duration of nutritional therapy, and length of stay, none of the estimated coefficients reached statistical significance. Overall, these findings suggest that the DRG reform did not substantially alter nutritional therapy expenditures or resource utilization patterns among the resident insurance population.

In contrast, the employee insurance subgroup showed more pronounced changes after policy implementation. Enteral nutrition costs exhibited a significant upward baseline trend (β_1_ = 6.999, *p* = 0.025), a significant immediate level increase at implementation (β_2_ = 461.536, *p* = 0.022), followed by a significant downward slope change (β_3_ = −9.465, *p* = 0.020), indicating an initial surge in expenditure that subsequently reversed into a sustained decline. Parenteral nutrition costs showed no significant pre-policy trend (β_1_ = 26.254, *p* = 0.204), but a significant immediate reduction (β_2_ = −2861.593, *p* = 0.004) occurred following the reform, with no significant post-policy slope change (β_3_ = −3.725, *p* = 0.873). Total hospitalization costs increased significantly in the pre-intervention period (β_1_ = 271.014, *p* = 0.098), experienced a significant immediate level increase (β_2_ = 17,730.398, *p* = 0.016), and subsequently declined significantly over time (β_3_ = −537.787, *p* = 0.003). Surgical fees demonstrated a significant downward baseline trend (β_1_ = −32.745, *p* = 0.008), a marginally non-significant level decrease (β_2_ = −1430.260, *p* = 0.111), and a significant upward slope change (β_3_ = 62.817, *p* < 0.001). Duration of nutritional therapy showed a marginally significant upward pre-policy trend (β_1_ = 0.086, *p* = 0.074), with no significant changes at or after implementation. Length of stay exhibited a significant upward baseline trend (β_1_ = 0.084, *p* = 0.047), but no significant level or slope changes.

Taken together, the subgroup analyses reveal substantial heterogeneity in policy effects across insurance types. While the DRG reform had limited impact on patients with resident insurance, it generated significant and sustained changes in both nutritional therapy expenditures and hospitalization costs among patients with employee insurance, suggesting that the reform’s effects were concentrated among the latter population.

To formally assess whether policy effects differed by insurance type, we conducted interaction analyses by including interaction terms between the policy indicators and insurance type (Table 8).

The results showed that the interaction terms for total hospitalization costs (β = 117,775.389, *p* = 0.038) and length of stay (β = 43.263, *p* = 0.005) were statistically significant, indicating that the impact of DRG reform differed across insurance groups for these outcomes.

For parenteral nutrition costs and surgical fees, the interaction terms were marginally significant (*p* < 0.10), suggesting potential heterogeneity. In contrast, no significant interaction effects were observed for enteral nutrition costs or duration of nutritional therapy.

These results provide complementary evidence to the subgroup analyses and offer a more formal statistical test of heterogeneity.

### 3.4. Model Diagnostics for Serial Correlation

To assess the presence of serial correlation in the ITS models, we conducted Durbin–Watson (DW) and Breusch–Godfrey (BG) tests for all outcome variables. The DW statistics were generally close to 2, and the BG test results were not statistically significant, indicating no strong evidence of autocorrelation in the residuals (Table 9). These findings suggest that the model estimates and statistical inferences are unlikely to be biased by serial correlation.

### 3.5. Robustness Checks

To assess the sensitivity of our main findings to alternative model specifications, we conducted two robustness checks. First, we performed a placebo test by shifting the policy implementation date to January 2023, a time point at which no actual policy change occurred. Second, we excluded the three-month post-policy buffer period (January to March 2022) to evaluate whether the initial implementation phase disproportionately influenced the estimates.

#### 3.5.1. Robustness Check by Altering the Policy Implementation Date

To test whether the observed effects were attributable to the actual DRG implementation rather than coincidental temporal trends, we conducted a placebo test by artificially setting the policy intervention date to January 2023. All other model specifications remained unchanged, including the same set of control variables and the interrupted time series framework. A statistically significant coefficient under this false specification would suggest that the main findings may reflect secular trends or external shocks rather than the true policy effect.

As shown in Table 10, when the policy implementation date was artificially shifted to January 2023, none of the outcome variables exhibited statistically significant level changes or slope changes. These null findings suggest that the significant policy effects observed in the main analysis were not artifacts of secular temporal trends.

#### 3.5.2. Robustness Check by Excluding the Post-Policy Buffer Period

To examine whether the initial implementation period—during which clinical practices and administrative processes may have been adjusting—disproportionately influenced the estimated policy effects, we excluded the first three months following DRG implementation (January to March 2022) from the analytical sample. The ITS model was then re-estimated on the remaining observations to assess whether the main findings remained consistent.

Table 11 presents the ITS results after excluding the three-month post-policy buffer period. The pattern of findings closely mirrored that of the main analysis. Enteral nutrition costs continued to show a significant upward baseline trend (β_1_ = 5.755, *p* = 0.028), a marginally significant immediate level increase (β_2_ = 405.552, *p* = 0.054), and a significant downward slope change (β_3_ = −7.874, *p* = 0.041). Total hospitalization costs exhibited a significant upward baseline trend (β_1_ = 426.872, *p* = 0.003), a significant immediate level increase (β_2_ = 16,994.740, *p* = 0.022), and a significant downward slope change (β_3_ = −635.510, *p* < 0.001). Surgical fees demonstrated a significant downward baseline trend (β_1_ = −40.686, *p* < 0.001) and a significant upward slope change (β_3_ = 59.328, *p* < 0.001), with the immediate level change remaining non-significant (β_2_ = −752.721, *p* = 0.443). For parenteral nutrition costs, a significant immediate level decrease was sustained (β_2_ = −3220.761, *p* = 0.001), consistent with the main findings.

Together, these sensitivity analyses support the robustness of the main ITS estimates to alternative model specifications.

### 3.6. Summary of Results 

The interrupted time series analysis revealed heterogeneous effects of the DRG policy across outcome measures. Following policy implementation, enteral nutrition costs exhibited a significant immediate increase followed by a sustained downward trend. Total hospitalization costs followed a similar pattern, with a sharp initial rise and a subsequent significant decline. Surgical fees demonstrated a significant downward baseline trend and a significant upward slope change post-implementation. Parenteral nutrition costs showed a significant immediate reduction without a subsequent trend change. In contrast, the duration of nutritional therapy and length of stay did not show statistically significant changes in all of the estimated parameters.

Subgroup analyses by type of medical insurance suggested heterogeneity in effects. Among patients covered by Urban Employee Basic Medical Insurance, the estimated policy effects were generally consistent with those observed in the main analysis, with significant changes in several outcome measures including enteral nutrition costs, total hospitalization costs, surgical fees, and parenteral nutrition costs. In contrast, patients with Urban and Rural Resident Basic Medical Insurance did not show statistically significant effects across most outcome measures, suggesting that observed policy effects were mainly driven by the employee insurance group.

## 4. Discussion

This study is the first to apply an interrupted time series framework to assess how the implementation of DRG-based payment reform influences nutritional therapy costs among gastric cancer surgery patients in China. By leveraging a quasi-experimental design, we were able to disentangle pre-existing trends from policy-induced changes, providing robust evidence on the dynamic effects of the reform across multiple outcome measures.

### 4.1. The Impact of DRG Reform on Nutritional Therapy Expenditures

Prior to DRG implementation, enteral nutrition costs showed a significant upward trend (β_1_ = 5.803, *p* = 0.027), indicating an average increase of approximately 5.8 RMB units per time period. This suggests a steady increase in enteral nutrition expenditure, which may reflect increased attention to perioperative nutritional support [16]. Under the fee-for-service model, such growth may also reflect provider responses to reimbursement incentives.

At the onset of DRG reform, enteral nutrition expenditure increased sharply (β_2_ = 395.703, *p* = 0.032), corresponding to an immediate rise of nearly 396 currency units. This magnitude indicates a substantial short-term escalation. Clinically, this may reflect short-term adjustments in clinical practice under the new payment system [13,30]. This short-term increase is consistent with evidence from DRG reforms showing that providers often exhibit transitional behavioral responses—including temporary increases in specific services and adjustments in coding or treatment patterns—before longer-term cost-containment effects emerge [20,33,34]. Over time, a significant negative slope change was observed (β_3_ = −7.778, *p* = 0.032), indicating that the post-intervention trend decreased by 7.8 units per period relative to the pre-intervention trend. Given a pre-intervention increase of 5.8 units per period (β_1_ = 5.803), this implies that the trend reversed from an upward trajectory to a gradual decline (approximately −2.0 units per period) after DRG implementation. Prior studies have shown that institutions often adjust resource allocation and limit non-essential services in response to reimbursement changes [35]. This pattern is consistent with a growing body of literature showing that DRG reforms trigger dynamic provider responses [36], including short-term adjustments in service utilization and treatment patterns, followed by longer-term cost containment and resource reallocation as financial incentives become binding. The initial increase may reflect a transitional response following policy implementation. However, whether reduced enteral nutrition has implications for patient recovery and quality of life remains an important question for future research.

In contrast, parenteral nutrition costs showed no significant baseline trend (β_1_ = 22.984, *p* = 0.188) and no significant slope change (β_3_ = −0.131, *p* = 0.995), indicating relative stability in long-term utilization. However, a significant immediate reduction was observed (β_2_ = −2917.276, *p* = 0.001), corresponding to a decrease of nearly 2917 currency units at policy implementation. This large effect size suggests an abrupt adjustment, which may be related to cost-control incentives under DRG at the early stage of reform. The lack of sustained decline suggests that parenteral nutrition use remained relatively stable, particularly in patients with postoperative gastrointestinal dysfunction or complications [17]. This pattern is consistent with previous findings that clinically indispensable treatments are less responsive to DRG-induced cost-containment pressures compared with discretionary services. Even under cost-control policies, hospitals may need to balance clinical needs and cost considerations, resulting in only modest reductions in parenteral nutrition expenditures.

The duration of nutritional therapy did not change significantly after DRG implementation, suggesting that treatment time remained stable despite financial pressure. This suggests that cost reductions may be associated with changes in prescribing patterns, product selection, or unit prices, rather than reductions in overall service volume. For gastric cancer patients, nutritional support remains an important component of perioperative care [18]. Therefore, maintaining treatment duration while controlling costs may reflect changes in practice under cost-containment incentives, although the implications for care quality cannot be determined from the current data.

To address whether changes in nutritional costs were driven by price, utilization frequency, or service composition, we further interpreted the ITS results in the context of DRG payment characteristics. DRG reform primarily constrains total expenditure rather than unit prices of specific nutritional products. Thus, observed changes in enteral and parenteral nutrition costs may be related to changes in utilization patterns rather than price effects, although this cannot be directly determined from the available data. Enteral nutrition cost showed an immediate increase followed by a declining trend, suggesting short-term higher utilization with subsequent adjustment. In contrast, parenteral nutrition cost showed a significant immediate reduction without further trend change (β_2_ = −2917.276, *p* = 0.001), suggesting a reduction in use or possible substitution effect.

### 4.2. The Impact of DRG Reform on Total Hospitalization Costs and Surgical Fees

Total hospitalization costs exhibited a significant upward pre-intervention trend (β_1_ = 431.584, *p* = 0.003), indicating an average increase of approximately 432 currency units per period. This reflects rising healthcare expenditures prior to reform. At the time of DRG implementation, there was a substantial immediate increase (β_2_ = 15,959.403, *p* = 0.019), equivalent to nearly 15,959 currency units. This large effect size suggests a pronounced short-term rise in hospital spending, which may reflect short-term adjustment following policy implementation [33]. Following implementation, a significant negative slope change was observed (β_3_ = −632.069, *p* < 0.001), indicating that the post-intervention trend decreased by approximately 632 units per period relative to the pre-intervention trend. Given the pre-intervention upward trend (β_1_ = 431.584), this implies that the trajectory of total hospitalization costs shifted from a steady increase to a gradual decline (approximately −200.5 units per period) after DRG reform. This finding suggests that DRG implementation was associated with changes in the trajectory of hospitalization costs. In practical terms, this suggests sustained cost containment over time, with cumulative reductions that may offset the initial increase observed at policy onset. This dynamic pattern is consistent with prior evidence on medium- and long-term DRG effects [34].

Surgical fees demonstrated a significant downward baseline trend (β_1_ = −39.951, *p* < 0.001), a marginally significant immediate level decrease (β_2_ = −1442.363, *p* = 0.085), and a significant upward slope change (β_3_ = 68.107, *p* < 0.001). This complex pattern suggests that while surgical costs were already declining before the reform, the post-policy period saw a gradual increase in the trajectory. This contrasts with the dynamic pattern observed for enteral nutrition costs, which showed an initial spike followed by a clear downward trend, indicating a transition from short-term adaptation to gradual adjustment over time. The sustained downward trend in total hospitalization costs, coupled with the gradual upward shift in surgical fees, may reflect changes in the composition of hospital expenditures under DRG constraints. Rather than reducing all categories of expenditure uniformly, different categories of expenditure showed divergent trends after the reform. This suggests that DRG reform may alter the internal composition of hospital costs, not only the overall level of expenditure.

### 4.3. Heterogeneity by Type of Medical Insurance

Subgroup analyses revealed substantial heterogeneity in policy effects across insurance types. Among patients covered by Urban Employee Basic Medical Insurance, the policy effects largely mirrored those observed in the main analysis. In contrast, patients with Urban and Rural Resident Basic Medical Insurance exhibited no significant policy effects across most outcome measures. The only notable finding was a significant upward baseline trend for total hospitalization costs (β_1_ = 872.634, *p* = 0.010), with no significant level or slope changes following implementation.

This heterogeneity may be related to differences in reimbursement rates, institutional incentives, and patient characteristics across the two insurance schemes. Urban Employee Basic Medical Insurance typically offers higher reimbursement rates and covers a broader range of services, making hospitals more responsive to payment changes for this population [37]. In contrast, the lower reimbursement rates associated with resident insurance may create weaker financial incentives for hospitals to alter clinical practices. From a health economics perspective, these differences can be further understood through the lens of reimbursement incentives, patient cost-sharing, and the principal–agent problem. Under Urban Employee Basic Medical Insurance, higher reimbursement rates and lower out-of-pocket payments may strengthen both patient demand for services and providers’ incentives to supply reimbursable care, amplifying behavioral responses to DRG payment changes. In contrast, under Urban and Rural Resident Basic Medical Insurance lower reimbursement levels and higher patient cost-sharing may constrain both demand and supply, thereby dampening the observable effects of the reform. Additionally, patients with employee insurance may have more complex clinical profiles or greater access to advanced treatments, rendering them more sensitive to policy-induced changes in resource allocation. This pattern may also reflect cost-shifting behavior, whereby providers respond more actively to higher-reimbursed patient groups. These findings underscore the importance of designing differentiated incentive mechanisms tailored to institutional and population-specific contexts. These interpretations should be viewed in light of the descriptive nature of the analysis.

Nevertheless, given the relatively small sample size in the employee insurance group, these interaction results should be interpreted with caution, and further validation with larger samples is warranted.

### 4.4. Policy Implications

These findings have several implications for DRG design and implementation. First, DRG payment standards should distinguish between clinically necessary nutritional support and potentially discretionary use of nutritional agents, particularly among high-risk surgical patients. Second, monitoring systems should prioritize both expenditure trends and indicators of care processes, such as nutritional screening and appropriate nutritional support, under case-based payment. Third, DRG grouping and reimbursement rules should account for patient complexity and insurance type to avoid unintended under-provision of supportive care among vulnerable groups. Finally, hospitals should develop evidence-based nutritional therapy pathways to ensure that cost containment is achieved through efficiency improvement rather than simple reduction in service use.

### 4.5. Limitations and Future Directions

Several limitations should be acknowledged. First, this study is limited to a single disease category within one city, which may not reflect the reform’s broader impact across different clinical contexts or geographic regions. The effectiveness of DRG payment reforms in controlling costs can vary across healthcare systems. In more developed regions, hospitals benefit from stronger managerial capabilities and higher levels of digital infrastructure, allowing them to leverage DRG mechanisms to improve efficiency and reduce overall expenditures. Conversely, in under-resourced areas, the implementation of DRG reforms may face significant barriers, including poor data quality and limited information systems, making it difficult for payment standards to reflect real-world treatment costs accurately [38].

Second, this study may be subject to selection bias related to the inclusion of patients. Because of limitations in the retrospective clinical database, we were unable to identify patients who had indications for nutritional therapy but did not receive it during hospitalization. As a result, the study only included patients with documented and standardized nutritional treatment. This may introduce selection bias, as patients who did not receive nutritional therapy—despite potential clinical indications—were not captured in the dataset. These patients may differ systematically in disease severity, nutritional risk, or treatment decision-making. As a result, the study sample may overrepresent patients with more standardized or clinically indicated nutritional therapy, while underrepresenting those with borderline indications or discretionary use. This could lead to an underestimation of the policy’s impact on overall nutritional resource allocation, particularly if DRG reform affected the decision to initiate nutritional therapy. Therefore, the estimated effects should be interpreted as reflecting changes among treated patients, rather than the full population of patients with nutritional needs. This limitation is inherent to the available data and could not be addressed in the current analysis.

Third, while our ITS design accounts for pre-existing trends and policy-induced changes, we cannot rule out the influence of concurrent policy initiatives, such as centralized drug procurement programs, which may have interacted with DRG implementation to shape the observed patterns. Future studies should employ multi-site designs and longer follow-up periods to capture the full trajectory of policy effects.

Fourth, the current analysis focused on costs and resource utilization, without direct assessment of clinical outcomes. Reduced nutritional therapy expenditures may reflect changes in resource use; therefore, whether such changes have implications for patient recovery, nutritional status, or quality of life cannot be determined from the current data and remains an important area for future research. In addition, the Urban Employee Basic Medical Insurance subgroup was relatively small, particularly in the post-intervention period; therefore, subgroup estimates should be interpreted cautiously and confirmed in larger multi-center datasets.

## 5. Conclusions

This study leverages a natural experiment from a 2022 DRG payment reform in a major Chinese city to evaluate its impact on nutritional therapy expenditures and healthcare utilization among gastric cancer surgery patients using inpatient data from 2018 to 2024. The results show that the reform significantly reshaped expenditure patterns, with enteral nutrition costs exhibiting an initial surge followed by a sustained decline, total hospitalization costs decreasing significantly over time, and parenteral nutrition costs showing an immediate reduction without a subsequent trend change. Heterogeneity analyses revealed that these effects were concentrated among patients covered by Urban Employee Basic Medical Insurance, whereas those with Urban and Rural Resident Basic Medical Insurance experienced no significant policy impacts. Notably, the duration of nutritional therapy and length of stay did not exhibit sustained changes, with no substantial changes observed in these measures. Robustness checks, including a placebo test and exclusion of the post-policy buffer period, supported the validity of the main findings. The reform’s effects demonstrated a dynamic pattern of a dynamic pattern characterized by an initial change followed by a gradual trend shift, underscoring the need for continuous policy monitoring, tailored strategies for heterogeneous insurance populations, and the integration of nutritional support guidelines into DRG frameworks to support cost containment and highlight the need for further evaluation of clinical and economic outcomes.

## Figures and Tables

**Table 1:** Study Variables and Definitions.

Category	Variable	Definition	Measurement/Assignment Description
Nutritional Therapy Cost and Intensity	Enteral Nutrition Cost	Total cost of enteral nutrition formulations during hospitalization (RMB)	Continuous variable; extracted from the hospital billing system; includes costs of enteral nutrition formulations only; excludes administration-related costs such as tubing and nursing services; unit: RMB.
	Parenteral Nutrition Cost	Total cost of parenteral nutrition formulations during hospitalization (RMB)	Continuous variable; extracted from the hospital billing system; includes parenteral nutrition formulations only; excludes administration-related costs; unit: RMB.
	Duration of Nutritional Therapy	Duration of nutritional therapy during hospitalization	Continuous variable; calculated as (date of last administration − date of first administration + 1), measured in days.
Healthcare Resource Utilization	Total Hospitalization Cost	Total direct medical cost during hospitalization (RMB)	Continuous variable; extracted from the hospital billing system; includes bed fees, diagnostic tests, medications, surgical procedures, medical consumables, and nursing services; unit: RMB.
	Surgical fees	Medical expenses related to surgical procedures during this hospitalization (RMB)	Continuous variable; extracted from the hospital billing system; includes fees directly related to surgical procedures; excludes medication, consumables, and anesthesia costs; unit: RMB.
	Length of stay	Actual number of days the patient stayed during this hospitalization	Continuous variable; calculated as (discharge date − admission date + 1), measured in days.
Interrupted Time Series Variables	Time (t)	Continuous time variable indicating monthly sequence of observations	Continuous variable; constructed as sequential month count from the start of the study period (t = 1, 2, …, T).
	Policy Indicator (post)	Indicator for policy implementation period	Binary variable; 0 = pre-intervention period, 1 = post-intervention period (January 2022 onward).
	Post-intervention Time Trend (post_trend)	Interaction term capturing change in trend after policy implementation	Continuous variable; calculated as t × post.
Control Variables	Age	Patient’s age at admission in completed years	Continuous variable; age at admission measured in completed years (years).
	Sex	Patient’s biological sex	Categorical variable; coded as 0 = female, 1 = male based on medical record information.
	Type of Medical Insurance	Type of medical insurance used by the patient during this hospitalization	Categorical variable; originally recorded in the hospital information system and recoded as 1 = Urban Employee Basic Medical Insurance, 2 = Urban and Rural Resident Basic Medical Insurance.
	Preoperative Prealbumin	Serum concentration of prealbumin from the last test before surgery	Continuous variable; serum prealbumin level measured at the last available laboratory test prior to surgery; unit: mg/L.
	BMI	Body Mass Index, an objective indicator for assessing nutritional status	Continuous variable; calculated as body weight (kg) divided by height squared (m^2^), measured at admission.
	Type of Surgery	Category of surgery performed during this hospitalization	Categorical variable; classified into four mutually exclusive categories (“radical”, “palliative”, “radical + palliative”, and “other”) based on predefined keyword classification rules applied to surgical procedure records in the electronic medical system.

**Table 2:** Baseline characteristics of the study population [n (%)].

Variable	Category	Pre-Intervention (*n* = 261)	Post-Intervention (*n* = 500)	Total (*n* = 761)
Sex	Female	82 (31.4)	130 (26.0)	212 (27.9)
	Male	179 (68.6)	370 (74.0)	549 (72.1)
Age	<60 years	58 (22.2)	145 (29.0)	203 (26.7)
	≥60 years	203 (77.8)	355 (71.0)	558 (73.3)
Insurance Type	Urban Employee Basic Medical Insurance	71 (27.2)	16 (3.2)	87 (11.4)
	Urban–Rural Resident Basic Medical Insurance	190 (72.8)	484 (96.8)	674 (88.6)
Surgery type	Radical	106 (40.6)	35 (7.0)	141 (18.5)
	Palliative	15 (5.7)	18 (3.6)	33 (4.3)
	Radical + Palliative	1 (0.4)	2 (0.4)	3 (0.4)
	Other	139 (53.3)	445 (89.0)	584 (76.7)
Nutritional therapy	Combined	210 (80.5)	400 (80.0)	610 (80.2)
	Enteral only	5 (1.9)	30 (6.0)	35 (4.6)
	Parenteral only	46 (17.6)	70 (14.0)	116 (15.2)

Note: “Radical + Palliative” indicates patients who underwent both radical and palliative procedures during the same hospitalization.

**Table 3:** Comparison of Baseline Continuous Variables Between Pre- and Post-intervention Groups.

Variable	Pre-Intervention (*n* = 261)	Post-Intervention (*n* = 500)	Total (*n* = 761)	*p*
Age (years)	66.0 (61.0, 72.0)	66.0 (58.0, 72.0)	66.0 (59.0, 72.0)	0.299
BMI (kg/m^2^)	23.39 (21.15, 25.20)	23.38 (21.10, 25.39)	23.38 (21.10, 25.39)	0.682
Preoperative prealbumin (mg/L)	190.0 (160.0, 228.2)	197.7 (159.4, 237.2)	195.9 (160.0, 230.3)	0.462

Note: Data are presented as median (interquartile range), M (Q_1_, Q_3_).

**Table 4:** Comparison of Outcome Variables Before and After DRG Implementation.

Indicator	Before DRG Implementation	After DRG Implementation	Z	*p*
Enteral nutrition cost	403.10 (156.54, 708.97)	610.49 (167.79, 951.91)	−3.785	<0.001
Parenteral nutrition cost	3353.21 (2109.09, 5385.18)	1827.31 (878.64, 3165.62)	9.520	<0.001
Duration of nutritional therapy (days)	11.0 (8.0, 15.0)	10.0 (7.0, 14.0)	2.053	0.040
Total hospitalization cost	81,987.67 (68,938.50, 96,668.36)	74,014.10 (65,880.06, 83,041.13)	5.333	<0.001
Surgical fees	5807.63 (4975.61, 6914.57)	7798.62 (6874.84, 8323.69)	−12.625	<0.001
Length of stay	17.0 (14.0, 21.0)	16.0 (14.0, 20.0)	2.182	0.029

Note: Data are presented as median (M) and quartiles (Q_1_, Q_3_). A *p* value of <0.05 was considered statistically significant.

**Table 5:** Interrupted Time Series Analysis of Outcome Variables Before and After DRG Implementation.

Indicator	Baseline Trend		Level Change		Slope Change	
	β_1_	*p*	β_2_	*p*	β_3_	*p*
Enteral nutrition cost	5.803 ** (0.675, 10.931)	0.027	395.703 ** (34.194, 757.212)	0.032	−7.778 ** (−14.869, −0.687)	0.032
Parenteral nutrition cost	22.984 (−11.285, 57.252)	0.188	−2917.276 *** (−4686.661, −1147.892)	0.001	−0.131 (−40.212, 39.951)	0.995
Duration of nutritional therapy	0.055 (−0.031, 0.142)	0.208	0.216 (−4.282, 4.714)	0.925	−0.053 (−0.158, 0.052)	0.322
Total hospitalization cost	431.584 *** (147.950, 715.219)	0.003	15,959.403 ** (2670.989, 29,247.817)	0.019	−632.069 *** (−953.537, −310.600)	0.000
Surgical fees	−39.951 *** (−60.411, −19.490)	0.000	−1442.363 * (−3083.708, 198.981)	0.085	68.107 *** (38.687, 97.526)	0.000
Length of stay	0.060 (−0.021, 0.140)	0.147	−0.632 (−4.735, 3.471)	0.762	−0.043 (−0.140, 0.054)	0.386

Note: Data are presented as coefficients with confidence intervals in parentheses. *** *p* < 0.01, ** *p* < 0.05, * *p* < 0.1.

**Table 6:** ITS Regression Results for Outcome Variables: Urban and Rural Resident Basic Medical Insurance Subgroup.

Indicator	Baseline Trend		Level Change		Slope Change	
	β_1_	*p*	β_2_	*p*	β_3_	*p*
Enteral nutrition cost	4.181 (−7.360, 15.722)	0.472	1702.567 (−4604.734, 8009.868)	0.591	−33.748 (−158.120, 90.624)	0.589
Parenteral nutrition cost	19.099 (−46.515, 84.713)	0.563	−18,451.303 (−51,565.721, 14,663.114)	0.270	302.316 (−341.544, 946.176)	0.352
Duration of nutritional therapy	−0.005 (−0.212, 0.203)	0.964	−11.198 (−98.908, 76.512)	0.799	0.161 (−1.558, 1.880)	0.852
Total hospitalization cost	872.634 ** (214.793, 1530.474)	0.010	−101,551.449 (−352,219.108, 149,116.210)	0.421	1228.840 (−3561.247, 6018.927)	0.610
Surgical fees	−57.025 ** (−99.677, −14.373)	0.010	−27,000.000 (−64,181.766, 10,117.454)	0.151	581.328 (−145.150, 1307.805)	0.115
Length of stay	0.023 (−0.182, 0.228)	0.821	0.830 (−59.236, 60.897)	0.978	−0.093 (−1.280, 1.094)	0.876

Note: Data are presented as coefficients with confidence intervals in parentheses. ** *p* < 0.05.

**Table 7:** ITS Regression Results for Outcome Variables: Urban Employee Basic Medical Insurance Subgroup.

Indicator	Baseline Trend		Level Change		Slope Change	
	β_1_	*p*	β_2_	*p*	β_3_	*p*
Enteral nutrition cost	6.999 ** (0.875, 13.123)	0.025	461.536 ** (66.779, 856.294)	0.022	−9.465 ** (−17.405, −1.525)	0.020
Parenteral nutrition cost	26.254 (−14.304, 66.812)	0.204	−2861.593 *** (−4791.280, −931.907)	0.004	−3.725 (−49.546, 42.096)	0.873
Duration of nutritional therapy	0.086 * (−0.008, 0.181)	0.074	1.770 (−2.898, 6.438)	0.457	−0.092 (−0.203, 0.019)	0.103
Total hospitalization cost	271.014 * (−49.710, 591.738)	0.098	17,730.398 ** (3386.485, 32,074.311)	0.016	−537.787 *** (−897.784, −177.791)	0.003
Surgical fees	−32.745 *** (−56.841, −8.649)	0.008	−1430.260 (−3192.216, 331.696)	0.111	62.817 *** (30.669, 94.966)	0.000
Length of stay	0.084 ** (0.001, 0.167)	0.047	0.778 (−3.450, 5.006)	0.718	−0.076 (−0.174, 0.023)	0.133

Note: Data are presented as coefficients with confidence intervals in parentheses. *** *p* < 0.01, ** *p* < 0.05, * *p* < 0.1.

**Table 8:** Interaction Effects of DRG Reform by Insurance Type.

Indicator	Level Change Interaction Coefficient	*p*	Trend Change Interaction Coefficient	*p*
Enteral nutrition cost	1172.290 (−4192.280, 6536.859)	0.668	−23.013 (−127.484, 81.458)	0.665
Parenteral nutrition cost	24,457.939 * (−316.391, 492,32.269)	0.053	−481.160 * (−973.225, 10.904)	0.055
Duration of nutritional therapy	42.255 (−27.631, 112.142)	0.236	−0.829 (−2.190, 0.533)	0.232
Total hospitalization cost	117,775.389 ** (6403.080, 229,147.698)	0.038	−1696.102 (−3903.026, 510.822)	0.132
Surgical fees	29,839.208 * (−2007.132, 61,685.548)	0.066	−596.603 * (−1220.745, 27.539)	0.061
Length of stay	43.263 *** (12.777, 73.749)	0.005	−0.836 *** (−1.443, −0.229)	0.007

Note: Interaction terms were constructed between policy indicators (level and trend) and insurance type. Coefficients represent differential effects between insurance groups. *** *p* < 0.01, ** *p* < 0.05, * *p* < 0.1.

**Table 9:** Results of Durbin–Watson and Breusch–Godfrey serial correlation tests.

Indicator	Durbin–Watson	Breusch–Godfrey	*p*
Enteral nutrition cost	2.019	0.055	0.815
Parenteral nutrition cost	1.961	0.013	0.911
Duration of nutritional therapy	2.268	1.510	0.223
Total hospitalization cost	1.582	3.680	0.059
Surgical fees	1.795	0.806	0.372
Length of stay	2.198	0.787	0.378

**Table 10:** ITS Regression Results for Outcome Variables: Placebo Test with Policy Node Set to January 2023.

Indicator	Baseline Trend		Level Change		Slope Change	
	β_1_	*p*	β_2_	*p*	β_3_	*p*
Enteral nutrition cost	4.084 ** (0.847, 7.320)	0.013	688.544 (−266.839, 1643.927)	0.157	−10.357 (−23.217, 2.502)	0.114
Parenteral nutrition cost	13.929 (−28.849, 56.708)	0.523	−327.650 (−2212.978, 1557.679)	0.733	−28.972 (−76.592, 18.649)	0.233
Duration of nutritional therapy	−0.018 (−0.066, 0.030)	0.464	5.043 (−3.929, 14.016)	0.270	−0.062 (−0.184, 0.061)	0.323
Total hospitalization cost	38.277 (−109.034, 185.589)	0.610	−8536.972 (−29,281.556, 12,207.612)	0.419	−19.889 (−315.957, 276.180)	0.895
Surgical fees	11.393 (−3.894, 26.681)	0.144	774.330 (−1405.318, 2953.978)	0.486	1.030 (−30.816, 32.875)	0.949
Length of stay	−0.010 (−0.057, 0.037)	0.675	0.016 (−8.945, 8.977)	0.997	0.000 (−0.121, 0.121)	0.997

Note: Data are presented as coefficients with confidence intervals in parentheses. ** *p* < 0.05.

**Table 11:** ITS Regression Results for Outcome Variables: Excluding the Three-Month Buffer Period After Implementation.

Indicator	Baseline Trend		Level Change		Slope Change	
	β_1_	*p*	β_2_	*p*	β_3_	*p*
Enteral nutrition cost	5.755 ** (0.613, 10.897)	0.028	405.552 * (−6.620, 817.724)	0.054	−7.874 ** (−15.414, −0.334)	0.041
Parenteral nutrition cost	22.604 (−11.719, 56.928)	0.196	−3220.761 *** (−5186.615, −1254.907)	0.001	4.587 (−36.968, 46.142)	0.828
Duration of nutritional therapy	0.050 (−0.036, 0.137)	0.253	1.833 (−2.952, 6.618)	0.452	−0.071 (−0.177, 0.035)	0.190
Total hospitalization cost	426.872 *** (142.993, 710.750)	0.003	16,994.740 ** (2436.980, 31,552.500)	0.022	−635.510 *** (−966.554, −304.466)	0.000
Surgical fees	−40.686 *** (−61.274, −20.098)	0.000	−752.721 (−2676.996, 1171.554)	0.443	59.328 *** (27.223, 91.433)	0.000
Length of stay	0.057 (−0.024, 0.138)	0.170	0.557 (−3.809, 4.923)	0.802	−0.057 (−0.155, 0.042)	0.259

Note: Data are presented as coefficients with confidence intervals in parentheses. *** *p* < 0.01, ** *p* < 0.05, * *p* < 0.1.

## Data Availability

The datasets analyzed during the current study are not publicly available due to information security and confidentiality requirements, but are available from the corresponding authors on reasonable request.
